# Safety and efficacy of rivaroxaban versus warfarin in atrial fibrillation with stage 4 to 5 chronic kidney disease including dialysis

**DOI:** 10.1016/j.rpth.2026.103398

**Published:** 2026-02-17

**Authors:** Anwar Almajdi, Sara Almutairi, Maha Alharbi

**Affiliations:** 1 Ministry of Health, Clinical Pharmacy Department, Al-Jahra Hospital, Kuwait; 2 Kuwait University, College of Pharmacy, Pharmacy practice, Jabriya, Kuwait

**Keywords:** atrial fibrillation, chronic kidney disease, dialysis, rivaroxaban, warfarin

## Abstract

**Background:**

Patients with atrial fibrillation (AF) and advanced chronic kidney disease (CKD) face elevated risks of stroke and bleeding; yet, optimal anticoagulation remains uncertain due to their exclusion from randomized trials. This systematic review and meta-analysis evaluated rivaroxaban vs warfarin in patients with AF and moderate-to-advanced CKD including dialysis.

**Methods:**

We searched PubMed/MEDLINE, Embase, and Cochrane CENTRAL through August 2025 for observational studies comparing rivaroxaban with warfarin in adults with nonvalvular AF and CKD stages 4 to 5 including dialysis. Primary outcomes were stroke/systemic embolism and major bleeding. Pooled hazard ratios (HRs) were calculated using random-effects models. Risk of bias was assessed using ROBINS-I, and certainty of evidence was evaluated using GRADE.

**Results:**

Four observational studies encompassing 31,037 patients of whom 12,160 received rivaroxaban and 18,877 received warfarin. Mean age ranged from 66 to 80 years, with CHA_2_DS_2_-VASc scores ranging from 3.5 to 4.5. Reduced-dose rivaroxaban (10-15 mg daily) was commonly prescribed. Compared with warfarin, rivaroxaban demonstrated a 30% reduction in stroke/systemic embolism (pooled HR, 0.70; 95% CI, 0.54-0.92; *P* = .009; *I*^2^ = 38.1%) and 17% reduction in major bleeding (HR, 0.83; 95% CI, 0.72-0.97; *P* = .018). Favorable but nonsignificant trends were observed for intracranial hemorrhage (HR, 0.73; 95% CI, 0.49-1.08) and gastrointestinal bleeding (HR, 0.68; 95% CI, 0.46-1.03). Overall evidence quality was moderate according to GRADE assessment.

**Conclusion:**

In patients with AF and advanced CKD including dialysis, rivaroxaban may be associated with improved efficacy and safety compared with warfarin. However, heterogeneity in CKD stages and off-label dosing practices necessitate prospective randomized trials to establish definitive treatment recommendations.

## Essentials

• Patients with irregular heart rhythm and severe kidney disease need blood thinners but face high risks.
• This study analyzed outcomes among 32,845 patients on dialysis treated with rivaroxaban or warfarin using observational data.
• Rivaroxaban reduced stroke risk by 30% and major bleeding by 17% compared with warfarin.
• These findings suggest rivaroxaban may be safer and more effective, but trials are needed.

## 1 Introduction

Chronic kidney disease (CKD) is defined as the presence of abnormalities in kidney function or structure for at least 3 months [1]. The incidence of CKD is increasing, specifically in its advanced stages, making it a major cause of cardiovascular-related events and mortality [2]. CKD is classified according to the glomerular filtration rate (GFR) into 5 stages as follows—G1: GFR, ≥90 mL/min/1.73 m^2^; G2: GFR, 60-89 mL/min/1.73 m^2^; G3a: GFR, 45-59 mL/min/1.73 m^2^; G3b: GFR, 30-44 mL/min/1.73 m^2^; G4: GFR, 15-29 mL/min/1.73 m^2^; and G5: GFR, <15 mL/min/1.73 m^2^ [1]. Advanced CKD stages are G4 and G5.

Among patients with CKD, the incidence of atrial fibrillation (AF) is higher than in those without CKD [3]. AF is one of the most common forms of arrhythmia. According to the Global Burden of Disease 2021 study, more than 52 million individuals are affected with AF worldwide [4]. AF is associated with cardiovascular and cerebrovascular complications, including stroke, thromboembolism, and heart failure [5], thus increasing patient morbidity and mortality rates. Approximately 30% of patients diagnosed with AF also have stage 3 to 5 CKD [6]. Impaired renal function alters drug metabolism and increases bleeding risk, which complicates the clinical management of AF including the safe and effective use of anticoagulant therapy [3].

Oral anticoagulation remains the cornerstone for preventing thromboembolic events in patients with nonvalvular AF (NVAF). The introduction of direct oral anticoagulants (DOACs) has transformed anticoagulation management in the general AF population [7]. However, their optimal use in advanced CKD represents one of the most challenging and understudied areas in thrombosis management. Each DOAC has distinct pharmacokinetic properties that become particularly relevant in renal impairment. Rivaroxaban exhibits ∼33% renal elimination as unchanged drug [8], occupying an intermediate position between highly renally cleared agents, such as dabigatran (80%) and edoxaban (50%), and minimally renally cleared agents, such as apixaban (27%) [9]. This pharmacokinetic profile suggests that rivaroxaban may offer a balance between predictable drug exposure and manageable renal accumulation in advanced CKD.

Despite over a decade of clinical use, critical knowledge gaps persist regarding the safety and efficacy of rivaroxaban populations with advanced CKD. Although apixaban has been most frequently prescribed DOAC in patients with severe renal impairment in some regions, accounting for 10.4% vs 9.5% for rivaroxaban in US registry data [10], the evidence base for each DOAC must be evaluated independently. DOACs differ fundamentally in their pharmacokinetic properties, renal clearance pathways, dosing protocols, and the populations studied in clinical trials. These differences limit the use of safety and efficacy data from 1 agent to another, particularly in advanced CKD where drug metabolism is altered. Therefore, a comprehensive rivaroxaban-specific evidence is essential to inform clinical decision making, regardless of prescribing trends for other DOACs. DOACs in advanced CKD are fundamentally distinct and cannot be considered interchangeable. Drug-specific pharmacokinetic properties, dosing strategies, and patient populations studied differ significantly across agents, necessitating separate evaluation of each DOACs risk–benefit profile.

This systematic review and meta-analysis addresses a critical unmet need by providing the first comprehensive synthesis of rivaroxaban vs warfarin outcomes specifically in patients with NVAF and advanced CKD (stages 4-5) including dialysis. Our analysis is timely and clinically relevant for the underrepresentation of patients with advanced CKD in randomized controlled trials (RCTs).

## 2 Methods

This systematic review and meta-analysis were registered in PROSPERO (CRD420251102017) and reported according to the 2020 Preferred Reporting Instructions for Systematic Reviews and Meta-analysis (PRISMA) guidelines [11].

### 2.1 Search strategy

A comprehensive literature search was conducted across the following electronic databases: PubMed/MEDLINE, Embase, Cochrane CENTRAL, from inception to August 7, 2025. The search was restricted to studies published in English, involving human adults.

Search terms included combinations of atrial fibrillation, chronic kidney disease, CKD, dialysis, rivaroxaban, and warfarin. Gray literature sources (eg, clinicaltrials.gov) and reference lists of included studies and relevant reviews were also screened to identify additional eligible studies. Duplicate records were removed, and all screening was conducted using the Rayyan.ai software [12].

### 2.2 Inclusion and exclusion criteria

Observational studies conducted on adults aged 18 years or older with NVAF and specifically defined an estimated glomerular filtration rate (eGFR) of <30 mL/min/1.73 m^2^ or creatinine clearance of <30 mL/min, including patients on dialysis, were included. While our primary focus was advanced CKD (stages 4-5 and dialysis), we included studies with mixed CKD populations (stages 4-5) when stage-specific data extraction was not feasible, recognizing that this introduces heterogeneity in renal function severity across our pooled analysis. Eligible studies had to compare rivaroxaban (intervention) with warfarin (comparator) and report at least 1 of the following clinical outcomes: stroke or systemic embolism, major bleeding, intracranial haemorrhage (ICH), gastrointestinal bleeding (GIB), all-cause mortality, or cardiovascular-related mortality.

We included studies regardless of whether they reported CKD stage–stratified outcomes but prioritized extraction of stage-specific data when available to assess potential effect modification by renal function severity. For each study, we extracted the following: (1) CKD stage distribution or mean eGFR; (2) stage-specific outcome data when reported (outcomes stratified by eGFR categories or CKD stages); and (3) rivaroxaban dosing patterns by CKD stage when available.

Studies were excluded if they focused exclusively on valvular AF; were case reports, editorials, reviews, or commentaries without original data; or did not report renal function or provide subgroup analyses specific to the CKD population.

### 2.3 Data extraction

Three reviewers (A.M., S.A., and M.A.) independently screened titles, abstracts, and full texts using the Rayyan.ai software [12]. Discrepancies were resolved through discussion. A standardized data extraction form was used to collect information on the following: (1) study characteristics—authors, publication year, study design (prospective vs retrospective), country, sample size, follow-up duration, and matching/adjustment methods (propensity score matching, inverse probability weighting, and multivariable adjustment); (2) population characteristics—age, sex, comorbidities (diabetes, hypertension, prior stroke, and heart failure), baseline CHA_2_DS_2_-VASc score, HAS-BLED score, and bleeding history; and (3) CKD-specific data (critical for stage-stratified analysis): CKD stage distribution (percentage of patients in stages 3a, 3b, 4, 5 nondialysis, and 5 dialysis), mean or median eGFR overall and by treatment group, method of eGFR calculation, and dialysis modality (hemodialysis vs peritoneal dialysis) when specified.

### 2.4 Outcome definitions

The primary efficacy outcome of this meta-analysis was stroke or systemic embolism, and the primary safety outcome was major bleeding, defined according to the International Society on Thrombosis and Haemostasis (ISTH) criteria. Major bleeding included fatal bleeding, symptomatic bleeding in a critical area or organ, or bleeding leading to a hemoglobin decrease of ≥20 g/L or requiring transfusion of ≥2 units of blood [13]. The secondary outcomes reported were ICH and GIB.

### 2.5 Statistical analysis

#### 2.5.1 Overall pooled analysis

Meta-analysis was conducted using a DerSimonian–Laird random-effects model using R Meta package (R Foundation of Statistical Computing). Time-to-event outcomes were pooled using hazard ratios (HRs). All pooled estimates are reported with 95% CIs. Statistical heterogeneity was assessed using the *I*^2^ statistic, with thresholds of 25%, 50%, and 75% indicating low, moderate, and high heterogeneity, respectively. When substantial heterogeneity (*I*^2^ > 50%) was detected, we explored potential sources in discussion.

#### 2.5.2 Risk of bias assessment

Risk of bias was assessed using the ROBINS-I v2 tool for nonrandomized studies of interventions [14]. ROBINS-I evaluates bias across 7 domains, including confounding, participant selection, intervention classification, deviations from intended interventions, missing data, outcome measurement, and selective reporting. Each domain and overall risk of bias were judged as low, moderate, serious, or critical.

#### 2.5.3 Assessment of certainty in evidence

The GRADE (Grading of Recommendations, Assessment, Development, and Evaluation) approach was used to assess the certainty of the body of evidence for each outcome. This was evaluated across the following 5 domains: risk of bias, inconsistency, indirectness, imprecision, and publication bias. Certainty of evidence was categorized as high, moderate, low, or very low. A summary of findings was created as tables where appropriate [15].

## 3 Results

### 3.1 Study selection

**Figure 1:**
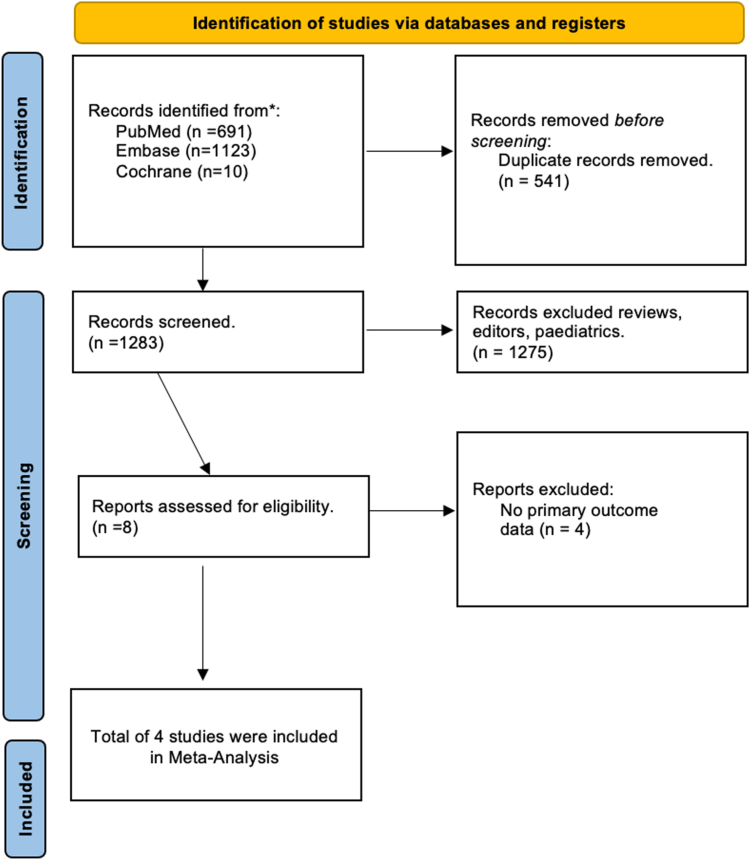
Preferred Reporting Instructions for Systematic Reviews and Meta-analysis (PRISMA) flow diagram.

A total of 1824 records were identified through database and additional source searches. After removing 541 duplicates, 1283 titles and abstracts were screened for title and abstract. Following this initial screening, full-text reviews were conducted for potentially eligible studies. Finally, 4 studies met the inclusion criteria, which were included [[16], [17], [18], [19]]. The study selection process is summarized in the PRISMA flow diagram (Figure 1).

### 3.2 Risk of bias assessment

The ROBINS-I tool found that the study by Ha et al. [17] had low risk of bias; that by Weir et al. [16] had moderate risk; and those by Coleman et al. [19] and Cheng et al. [18] had serious risk of bias.

### 3.3 Certainty of evidence

The overall quality of the included studies was generally moderate, with some studies rated as low or low to moderate (those by Ha et al. [17], Weir et al. [16], Coleman et al. [19], and Cheng et al. [18] were moderate) according to the GRADE approach. A full assessment for each study is provided in Supplementary.

### 3.4 Study characteristics

Four observational studies have evaluated rivaroxaban vs warfarin in patients with AF and moderate-to-advanced CKD [7,[16], [17], [18], [19]]. These studies, published between 2018 and 2024, were predominantly retrospective and conducted across diverse regions including the United States, Asia, and Australia/Canada. Study populations included patients with NVAF and CKD stages 3a to 5. However, the study by Ha et al. [17] also enrolled patients with CKD stage 2 (not individually specified but included in the stage 3a category, comprising 19.8% of their cohort). This broader inclusion, although introduced heterogeneity in renal function severity, reflected real-world clinical practice where anticoagulation decisions often must be made across the CKD spectrum with some including patients on dialysis and excluding those with end-stage renal disease. Sample sizes ranged from 347 to 6744 patients. Rivaroxaban dosing varied according to the renal function, with reduced doses (10-15 mg daily) commonly used in advanced CKD. Outcomes such as bleeding and stroke/systemic embolism were primarily identified using International Classification of Diseases, ninth/tenth revision, codes. Follow-up periods ranged from ∼1 to 7 years. Included studies characteristics are summarized in Table 1.

**Table 1:** Summary of included studies.

Author	Year	Design	Country	AF population	CKD stage	Sample size	Rivaroxaban, *n* (dose)	Warfarin (*n*)	Bleeding definition	Stroke/SE definition	Follow-up period
Coleman et al. [19]	2019	Retrospective	United States	NVAF (OAC naive)	4 or 5 or HD	6744	1896 (∼734 received < 20 mg)	4848	Cunningham algorithm (ICD-10 codes)	ICD-10 codes	1.4 (0.6-2.7) y (median)
Ha et al. [17]	2023	Retrospective	Australia and Canada	AF/atrial flutter	2–5 (dialysis excluded)	5723	685	5038	ICD-10 codes	ICD-10 codes	7 y
Weir et al. [16]	2020	Retrospective cohort	United States	NVAF	4-5 dialysis	2317	781	1536 (match-weighted *n* = 781)	ICD-9/10 codes	ICD-9/10	R: 389 d W: 370 d (mean)
Cheng et al. [18]	2021	Retrospective cohort	Taiwan	NVAF	5 dialysis	3358	173 (50.8% on 10 mg, 38.7% on 15 mg, 10.4% on 20 mg)	3185	ICD-9	ICD-9	R: 19.1 mo W: 27.4 mo (mean)

AF, atrial fibrillation; CKD, chronic kidney disease; HD, hemodialysis; ICD, International Classification of Diseases; NVAF, nonvalvular atrial fibrillation; OAC, oral anticoagulant; R, rivaroxaban; SE, systemic embolism; W, warfarin.

### 3.5 Baseline demographics

Baseline demographics of the included studies are summarized in Table 2. A total of 31,037 patients were included, of whom 12,160 received rivaroxaban and 18,877 received warfarin. Mean age across studies ranged from 66 to 80 years, with generally comparable age distributions between treatment groups within each study. Men comprised ∼40% to 61% of the study populations. The prevalence of diabetes patients ranged from 30% to 41%, whereas hypertension was highly prevalent in included studies, which was ∼80%-97%. Thromboembolic risk was moderate to high, with mean CHA_2_DS_2_-VASc scores ranging from 3.5 to 4.5, and bleeding risk was observed in accordance with HAS-BLED scores, including 31.3% of patients with HAS-BLED of ≥3 in the study by Ha et al. [16].

**Table 2:** Baseline characteristics.

Characteristic	Ha et al. [17]		Weir et al. [16]		Coleman et al. [19]		Cheng et al. [18]	
	R	W	R	W	R	W	R	W
*n*	9310	9308	781	1536	1896	4848	173	3185
Age (y), mean	74	74	79	79.9	72	72	75	69
Male (%)	54	54	39.5	40.7	NR	NR	55	51
Female (%)	46	46	60.5	59.3	NR	NR	45	49
Diabetes (%)	30.3	30.3	39.2	38.2	NR	NR	NR	NR
Hypertension (%)	NR	NR	94.6	93.8	NR	NR	NR	NR
CHA_2_DS_2_-VASc	∼3.5	∼3.5	4.50 ± 1.51	4.49 ± 1.48	NR	NR	NR	NR
HAS-BLED ≥3	31.3%	31.3%	3.50 ± 0.96	3.53 ± 1.00	NR	NR	NR	NR
CKD stages, *n* (%)								
CKD 4 (15-29 mL/min/1.73 m^2^)	611 (2.2)	611 (2.2)	635 (81.3)	631.5 (80.9)	228 (12)	582 (12)	—	—
CKD 5 nondialysis	62 (0.2)	61 (0.2)	28 (3.6)	29.5 (3.8)	1668 (88)	4266 (88)	—	—
CKD 5 dialysis	—	114 (14.6)	120 (15.4)	—	—	173 (100)	3185 (100)	—

CKD, chronic kidney disease; CHA_2_DS_2_-VASc, congestive heart failure, hypertension, age ≥ 75 y (2 points), diabetes mellitus, prior stroke/transient ischemic attack/systemic embolism (2 points), vascular disease, age 65-74 y, sex category (female); HAS-BLED, hypertension, abnormal renal and liver function, stroke, bleeding history or predisposition, labile international normalized ratio, elderly (>65 y), drugs, or alcohol; NR, not reported; R, rivaroxaban; W, warfarin.

### 3.6 Rivaroxaban dosing

Reduced-dose rivaroxaban was commonly prescribed, particularly among patients with impaired renal function. In the study by Cheng et al. [18], 50.8% received 10 mg, 38.7% received 15 mg, and 10.4% received 20 mg of rivaroxaban daily. In the studies by Coleman et al. [19] and Weir et al. [16], dosing was frequently reported as mixed population. In the study by Coleman et al. [19], 38.7% received <20 mg daily, while 61.3% received 20 mg of rivaroxaban daily. In the study by Weir et al. [16], 60.1% received 15 mg, 14.7% received 20 mg, and 21.1% received <15 mg of rivaroxaban, with dosing not documented in 4.1% of patients. In the study by Ha et al. [17], doses were not specified. However, reduced-dose rivaroxaban was used in 46% of patients of overall CKD advanced stages. Summarized dosing pattern is provided in Supplementary Data.

### 3.7 Primary outcomes

**Figure 2:**
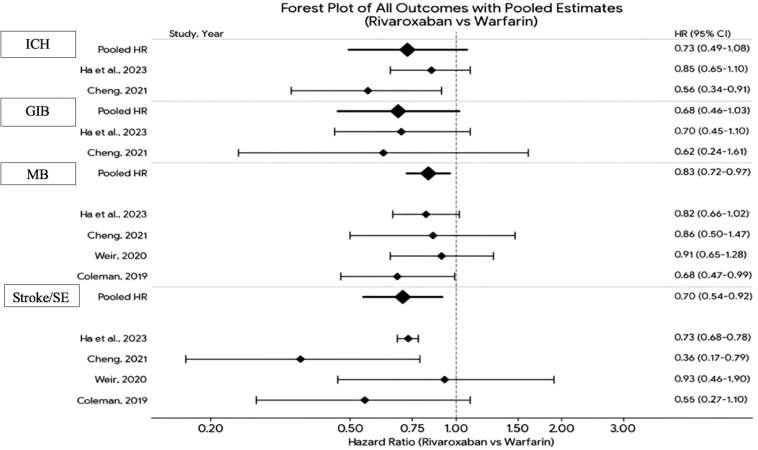
Forest plot of all outcomes with pooled estimates. GIB, gastrointestinal bleeding; HR, hazard ratio; ICH, intracranial hemorrhage; MB, major bleeding; SE, systemic embolism.

Rivaroxaban was associated with a statistically significant 30% reduction in the risk of stroke or systemic embolism compared with warfarin (pooled HR, 0.70; 95% CI, 0.54-0.92; *P* = .009). Statistical heterogeneity was low to moderate (*I*^2^ = 38.1%), indicating moderate consistency across studies. Rivaroxaban demonstrated a statistically significant 17% reduction in major bleeding risk compared with warfarin (pooled HR, 0.83; 95% CI, 0.72-0.97; *P* = .018), as shown in Figure 2.

### 3.8 Secondary outcomes

The pooled analysis demonstrated a 32% reduction in GIB risk with rivaroxaban (HR, 0.68; 95% CI, 0.46-1.03; *P* = .07), which was not statistically significant (Figure 2). No heterogeneity was detected (*I*^2^ = 0.0%; *P* = .82). Regarding ICH, the pooled analysis showed a 27% reduction in risk with rivaroxaban (HR, 0.73; 95% CI, 0.49-1.08; *P* = .12), which was also not statistically significant. Moderate statistical heterogeneity was observed (*I*^2^ = 53.4%) (Figure 2).

## 4 Discussion

This systematic review and meta-analysis of 6 observational studies encompassing 32,845 patients (13,173 receiving rivaroxaban and 19,672 receiving warfarin) with AF and moderate-to-advanced CKD including dialysis suggested that rivaroxaban may be associated with improved safety and efficacy outcomes compared with warfarin. Rivaroxaban significantly reduced the risk of stroke or systemic embolism by 30% (HR, 0.70; 95% CI, 0.54-0.92; *P* = .009) and major bleeding by 17% (HR, 0.83; 95% CI 0.72-0.97; *P* = .018). Although favorable trends were observed for ICH (27% reduction; HR, 0.73; 95% CI, 0.49-1.08) and GIB (32% reduction; HR, 0.68; 95% CI, 0.46-1.03), they did not reach statistical significance. These findings suggest a favorable net clinical benefit for rivaroxaban in this high-risk population.

Our findings extend previous evidence from ROCKET-AF trial: rivaroxaban demonstrated noninferiority to warfarin for stroke prevention in patients with AF, with a post hoc subgroup analysis suggesting consistent efficacy across the spectrum of renal function, although patients with GFR of <30 mL/min/1.73 m^2^ were excluded [20,21]. It showed comparable rates of stroke and systemic embolism between rivaroxaban and warfarin; however, major bleeding rates were numerically higher with rivaroxaban in this subgroup [21]. Nevertheless, our meta-analysis of real-world data demonstrated a more favorable safety profile, reflecting differences in patient selection, clinical practice patterns, or suboptimal anticoagulation control with warfarin. Warfarin management in patients with CKD is practically difficult, with multiple studies demonstrating poor time in therapeutic range, often 10% to 15% lower than that in patients with normal renal function, resulting in increased risks of both stroke and bleeding [22,23]. The study by Ha et al. [16], which represents the largest cohort in our analysis with 18,618 patients, demonstrated consistent safety and efficacy outcomes across CKD stages 3b to 5 excluding dialysis. In this study, rivaroxaban showed reduced risks of stroke/systemic embolism (HR, 0.73; 95% CI, 0.68-0.78), major bleeding (HR, 0.82; 95% CI, 0.66-1.02), ICH (HR, 0.85; 95% CI, 0.65-1.10), and GIB (HR, 0.70; 95% CI, 0.45-1.10) compared with warfarin across this broad spectrum of renal impairment. This real-world evidence is particularly important for patients with CKD, who are often excluded from RCTs due to concerns about bleeding risk and altered drug metabolism, creating a significant evidence gap for the very population that most needs effective anticoagulation [24,25]. However, for patients with advanced CKD stage 4 to 5 and those on dialysis, the systematic review and meta-analysis by Chen et al. [24] showed that rivaroxaban or apixaban are safe and at least as effective as warfarin in patients with AF and stage 4 to 5 CKD or on dialysis, supporting the inclusion of patients on dialysis in our pooled analysis [26].

A notable finding from our analysis is the high incidence of reduced-dose rivaroxaban across included studies, with 38.7% to 100% of patients receiving less than the standard 20-mg daily dose depending on the GFR. According to the prescribing information and regulatory approval of rivaroxaban, the recommended dose for patients with creatinine clearance of 15 to 49 mL/min is 15 mg. This reflects real-world adherence to dosing guidelines that recommend 15 mg daily for patients with GFR of 15 to 49 mL/min/1.73 m^2^ [27]. Cheng et al. [18] revealed particularly conservative dosing practices, with 50.8% of patients on dialysis receiving 10 mg daily and 38.7% receiving 15 mg daily, despite limited evidence for rivaroxaban use in end-stage renal disease [17]. Rivaroxaban is not approved for use in patients on dialysis, and both 10- and 15-mg dosing in this population represent off-label use. The 10-mg dose is not a licensed dose for any indication and appears to reflect clinical judgment aimed at minimizing drug accumulation in the absence of renal clearance. The rationale for these conservative dosing strategies originate from concerns about drug accumulation and bleeding risk, although the pharmacologic basis remains uncertain given high protein binding (92%-95%) of rivaroxaban, limiting dialytic removal. However, the appropriateness of these dosing strategies remains debated, although our pooled HR results suggest that the reduced dose administered in real-world practice achieve favorable outcomes [17,27].

Despite this dosing heterogeneity and off-label use, our pooled results suggest that the reduced doses of rivaroxaban administered in real-world practice, including the nonapproved 10-mg dose in dialysis, achieve favorable outcomes compared with warfarin. Moreover, from our data, whether outcomes differ between 10, 15, and 20mg doses could not be ascertained, as dose-stratified analyses were not possible. For patients with CKD stage 5 not on dialysis (GFR < 15 mL/min/1.73 m^2^), only 10 and 15 mg doses have been formally studied, creating a significant evidence gap. Our analysis included only 62 to 123 patients in this category (Table 2), insufficient to make definitive recommendations. Clinicians managing these patients must engage in shared decision making, weighing potential benefits against uncertain risks, with recognition that any dose used represents off-label prescribing.

The pharmacologic profile of rivaroxaban may offer advantages of use in CKD. Rivaroxaban undergoes ∼33% renal elimination in its unchanged form, with the remainder metabolized hepatically through CYP3A4/3A5 and CYP2J2 pathways [28,29]. This dual pathway of elimination provides some buffer against drug accumulation in renal impairment, although dose reduction remains necessary in moderate-to-severe CKD. Importantly, rivaroxaban offers the convenience of once-daily dosing even in patients with renal impairment (15 mg once daily for GFR of 15-49 mL/min/1.73 m^2^), which may enhance medication adherence compared with twice-daily regimens [29]. This once-daily regimen is particularly advantageous for patients with CKD who typically manage multiple comorbidities with polypharmacy, often with 10 to 15 concurrent medications, and may experience cognitive impairment or functional limitations that complicate medication adherence [30]. However, apixaban is also an effective option in patients with CKD with a lower renal elimination fraction (∼27%) and has demonstrated excellent outcomes in patients with renal impairment [31,32]. The once-daily dosing regimen of rivaroxaban presents a practical advantage, offering simplicity in clinical decision making, compared with apixaban’s weight- and creatinine-based dose adjustments (2.5 mg twice daily if 2 or more criteria are met: age ≥ 80 years, body weight ≤ 60 kg, or serum creatinine ≥ 1.5 mg/dL) [33,34].

Our analysis benefits from several methodological strengths that enhance confidence in the findings. The overall quality of included studies was rated moderate according to GRADE assessment, with most studies demonstrating a low to moderate risk of bias. The large sample size of 31,037 patients provides statistical power to detect clinically meaningful differences in both efficacy and safety outcomes, and the geographic diversity of included studies strengthens the generalizability of findings across different health care systems, prescribing patterns, and patient populations. In addition, the inclusion of patients across the full spectrum of CKD severity (stages 3a through 5, including patients on dialysis) allows our findings to inform treatment decisions across the wide range of renal impairment in clinical practice. Our analysis presented a comprehensive assessment of the benefit–risk profile evaluating multiple clinically relevant outcomes, including both thromboembolic events and various bleeding complications. The inclusion of predominantly real-world evidence from retrospective cohort studies and 1 prospective study provides valuable insights into clinical practice patterns and outcomes across diverse health care settings worldwide. This real-world perspective is particularly important in patients with CKD, who are often underrepresented in RCTs and may have different risk profiles than other trial populations.

The concept of net clinical benefit, weighing both thromboembolic and bleeding outcomes, is particularly relevant in the CKD population, where both risks are substantially elevated. Our meta-analysis extends this finding by showing simultaneous reductions in both stroke/systemic embolism (30% reduction) and major bleeding (17% reduction), suggesting a genuine improvement in the benefit–risk ratio rather than a trade-off between efficacy and safety.

The favorable bleeding profile observed with rivaroxaban deserves particular attention, as bleeding complications represent a major concern in patients with CKD. The pathophysiology of bleeding in CKD is multifactorial, involving uremic platelet dysfunction, altered von Willebrand factor metabolism, endothelial dysfunction, increased vascular calcification, anemia, and the frequent use of antiplatelet agents for concomitant cardiovascular disease [34,35]. Despite these intrinsic bleeding risks, rivaroxaban demonstrated a statistically significant 17% reduction in major bleeding compared with warfarin. The 27% reduction in ICH, although not reaching statistical significance, carries potential clinical importance given the high mortality rate (>50%) associated with this catastrophic complication in patients with CKD [36].

Based on the demonstrated dual benefit of stroke reduction and improved safety compared with warfarin, rivaroxaban may be considered a part of anticoagulation strategy in patients with advanced CKD and AF with appropriate reduced dosage. For patients with CKD stage 4 (GFR, 15-29 mL/min/1.73 m^2^), 15-mg once-daily rivaroxaban represents the appropriate evidence-based dose. However, in patients with stage 5 CKD (GFR <15 mL/min/1.73 m^2^) with or without dialysis, dosing remains more controversial due to limited prospective data, and individual risk–benefit assessment is needed. Accurate assessment of renal function is necessary as both overestimation and underestimation of GFR can lead to inappropriate dosing. Clinicians should use the Cockcroft–Gault equation consistently for dose determination, as this was used in the ROCKET-AF trial, and avoid switching between different GFR estimation methods [36]. Notably, the timing of rivaroxaban administration relative to dialysis sessions does not require adjustment, as minimal drug is removed during dialysis due to high protein binding [37].

Several important limitations warrant consideration. The observational nature of all included studies introduces potential for selection bias and residual confounding that adjustment methods cannot fully eliminate. Significant heterogeneity in renal function severity across our pooled population represents a major methodological limitation. The inclusion introduces heterogeneity that may reduce the ability to detect stage-specific effects. The optimal anticoagulant choice and dosing strategy likely differs substantially between patients with CKD stage 4 and those with CKD stage 5 on dialysis, yet our pooled analysis did not adequately distinguish between these clinically distinct populations. We were unable to perform meaningful subgroup analyses by specific CKD stages due to incomplete stage-stratified outcome reporting in primary studies. Heterogeneous rivaroxaban dosing patterns constitute another major source of potential confounding. Our analysis pooled patients receiving 10-, 15-, and 20-mg daily doses without stratification by the administered dose. These different dosing regimens may have distinct risk–benefit profiles, particularly in advanced CKD where drug accumulation becomes clinically relevant. The inability to perform dose-stratified analyses due to incomplete reporting substantially limits our capacity to make dose-specific recommendations. Moreover, a considerable proportion of patients received off-label doses, most notably 10 mg daily in patients on dialysis, and we could not determine whether the favorable outcomes observed reflect inherent safety of particular doses or careful patient selection by treating clinicians.

Our conservative methodological approach excluded studies enrolling mixed DOAC populations, even when rivaroxaban-specific subgroup data appeared extractable, which may have led to omission of valuable data, particularly for patients on dialysis. Critical deficiencies included absence of standardized outcome definitions, lack of reported HRs, small sample size (*n* = 347), and brief follow-up duration (16 ± 3 months). Outcome definitions varied across studies, with most relying on administrative International Classification of Diseases, ninth/tenth revision, codes for event ascertainment, which may lead to misclassification. Time in therapeutic range for warfarin patients was not consistently reported across studies, making it difficult to determine whether poor warfarin outcomes reflect suboptimal anticoagulation management or inherent pharmacologic limitations of warfarin in the CKD population. The appropriateness of prescribed rivaroxaban doses relative to actual renal function was not systematically evaluated in most studies, limiting our ability to separate drug effects from prescribing quality effects.

The evidence base for patients on dialysis, while representing the largest synthesis to date for this population, remains fundamentally limited. Only 287 rivaroxaban-treated patients on dialysis were included across 2 studies (Cheng et al. [18], *n* = 173; Weir et al. [16], *n* = 114), all receiving off-label doses without established safety or efficacy data. This modest sample size and observational design cannot provide definitive evidence for dialysis populations. These methodological limitations collectively indicate that although our findings provide valuable real-world insights supporting consideration of rivaroxaban as an alternative to warfarin in moderate-to-advanced CKD, they should not be interpreted as definitive evidence establishing superiority across all CKD stages or validating specific off-label dosing strategies. Prospective RCTs with careful CKD stage stratification, standardized dosing protocols, and adequate power remain urgently needed. The ongoing RENAL-AF trial comparing apixaban with warfarin in patients on dialysis may provide important insights; however, rivaroxaban-specific trials are lacking. These patients face a paradoxical situation—they have the highest risk of both thromboembolic stroke and bleeding complications, yet their complexity lead to their exclusion in most RCTs, creating a gap in guidelines and uncertainty regarding optimal anticoagulation strategy. Our meta-analysis provides important real-world insight that may help address this clinical dilemma.

## 5 Conclusion

This meta-analysis of real-world evidence suggests that rivaroxaban may be associated with reductions in both stroke/systemic embolism and major bleeding compared with warfarin, which provides a potential insight for clinicians managing AF in patients with advanced CKD, including those undergoing dialysis, although careful patient selection, accurate renal function assessment, and ongoing monitoring remain essential. This may represents a potential shift from warfarin use to reduced-dose rivaroxaban as a safe and effective alternative in this high-risk population.The evidence for patients on dialysis, while included in this analysis, remains less robust and requires further investigation through RCTs. Given the growing incidence of both AF and CKD in aging populations, optimizing anticoagulation strategies in this high-risk group represents a critical public health priority.
