# Supplementary material for: Safety and efficacy of rivaroxaban versus warfarin in atrial fibrillation with stage 4 to 5 chronic kidney disease including dialysis
Source: Res Pract Thromb Haemost. 2026 Feb 17;10(2):103398. doi: 10.1016/j.rpth.2026.103398 (PMC12992934; doi:10.1016/j.rpth.2026.103398)

AFCKD supplementary

Keywords

| Pubmed | (("Factor Xa Inhibitors"[Mesh] OR "Thrombin Inhibitors"[Mesh] OR rivaroxaban[tw] OR apixaban[tw] OR edoxaban[tw] OR dabigatran[tw] OR "direct oral anticoagulant*"[tw] OR "novel oral anticoagulant*"[tw] OR "new oral anticoagulant*"[tw] OR DOAC*[tw] OR NOAC*[tw] OR "factor Xa inhibitor*"[tw] OR "thrombin inhibitor*"[tw] OR xarelto[tw] OR eliquis[tw] OR savaysa[tw] OR lixiana[tw] OR pradaxa[tw])  AND  ("Warfarin"[Mesh] OR warfarin[tw] OR coumadin[tw] OR "vitamin K antagonist*"[tw] OR VKA*[tw] OR "vitamin K inhibitor*"[tw])  AND  ("Atrial Fibrillation"[Mesh] OR "atrial fibrillation"[tw] OR "atrial flutter"[tw] OR "non-valvular atrial fibrillation"[tw] OR NVAF[tw] OR AF[tw])  AND  ("Renal Insufficiency, Chronic"[Mesh] OR "Kidney Failure, Chronic"[Mesh] OR "Renal Dialysis"[Mesh] OR "chronic kidney disease"[tw] OR "chronic renal disease"[tw] OR "renal insufficiency"[tw] OR "kidney failure"[tw] OR "renal failure"[tw] OR "end-stage renal disease"[tw] OR ESRD[tw] OR CKD[tw] OR "severe renal impairment"[tw] OR "advanced CKD"[tw] OR dialysis[tw] OR hemodialysis[tw] OR haemodialysis[tw] OR "peritoneal dialysis"[tw] OR "stage 4 CKD"[tw] OR "stage 5 CKD"[tw] OR "GFR < 30"[tw] OR "eGFR < 60"[tw])) |
| --- | --- |
| Embase | (('factor xa inhibitor'/exp OR 'thrombin inhibitor'/exp OR 'rivaroxaban'/exp OR 'apixaban'/exp OR 'edoxaban'/exp OR 'dabigatran'/exp OR 'direct oral anticoagulant*':ab,ti,kw OR 'novel oral anticoagulant*':ab,ti,kw OR 'new oral anticoagulant*':ab,ti,kw OR doac*:ab,ti,kw OR noac*:ab,ti,kw OR 'factor xa inhibitor*':ab,ti,kw OR 'thrombin inhibitor*':ab,ti,kw OR xarelto:ab,ti,kw OR eliquis:ab,ti,kw OR savaysa:ab,ti,kw OR lixiana:ab,ti,kw OR pradaxa:ab,ti,kw)  AND  ('warfarin'/exp OR 'coumarin derivative'/exp OR warfarin:ab,ti,kw OR coumadin:ab,ti,kw OR 'vitamin k antagonist*':ab,ti,kw OR vka*:ab,ti,kw)  AND  ('atrial fibrillation'/exp OR 'atrial flutter'/exp OR 'atrial fibrillation':ab,ti,kw OR 'atrial flutter':ab,ti,kw OR 'non-valvular atrial fibrillation':ab,ti,kw OR nvaf:ab,ti,kw OR af:ab,ti,kw)  AND  ('chronic kidney failure'/exp OR 'kidney disease'/exp OR 'dialysis'/exp OR 'hemodialysis'/exp OR 'chronic kidney disease':ab,ti,kw OR 'chronic renal disease':ab,ti,kw OR 'renal insufficiency':ab,ti,kw OR 'kidney failure':ab,ti,kw OR 'renal failure':ab,ti,kw OR 'end-stage renal disease':ab,ti,kw OR esrd:ab,ti,kw OR ckd:ab,ti,kw OR 'severe renal impairment':ab,ti,kw OR 'advanced ckd':ab,ti,kw OR dialysis:ab,ti,kw OR hemodialysis:ab,ti,kw OR haemodialysis:ab,ti,kw OR 'peritoneal dialysis':ab,ti,kw OR 'stage 4 ckd':ab,ti,kw OR 'stage 5 ckd':ab,ti,kw OR 'gfr < 30':ab,ti,kw OR 'egfr < 30':ab,ti,kw)) |
| Cochrane | ((MeSH descriptor: [Factor Xa Inhibitors] explode all trees OR MeSH descriptor: [Thrombin Inhibitors] explode all trees OR (rivaroxaban or apixaban or edoxaban or dabigatran):ti,ab,kw OR ("direct oral anticoagulant*" or "novel oral anticoagulant*" or "new oral anticoagulant*"):ti,ab,kw OR (DOAC* or NOAC*):ti,ab,kw OR ("factor Xa inhibitor*" or "thrombin inhibitor*"):ti,ab,kw OR (xarelto or eliquis or savaysa or lixiana or pradaxa):ti,ab,kw)  AND  (MeSH descriptor: [Warfarin] explode all trees OR (warfarin or coumadin):ti,ab,kw OR ("vitamin K antagonist*" or VKA*):ti,ab,kw)  AND  (MeSH descriptor: [Atrial Fibrillation] explode all trees OR ("atrial fibrillation" or "atrial flutter"):ti,ab,kw OR ("non-valvular atrial fibrillation" or NVAF or AF):ti,ab,kw)  AND  (MeSH descriptor: [Renal Insufficiency, Chronic] explode all trees OR MeSH descriptor: [Kidney Failure, Chronic] explode all trees OR MeSH descriptor: [Renal Dialysis] explode all trees OR ("chronic kidney disease" or "chronic renal disease"):ti,ab,kw OR ("renal insufficiency" or "kidney failure" or "renal failure"):ti,ab,kw OR ("end-stage renal disease" or ESRD or CKD):ti,ab,kw OR ("severe renal impairment" or "advanced CKD"):ti,ab,kw OR (dialysis or hemodialysis or haemodialysis or "peritoneal dialysis"):ti,ab,kw OR ("stage 4 CKD" or "stage 5 CKD" or "GFR < 30" or "eGFR < 30"):ti,ab,kw)) |

**Demographics of included patients**

| **Study** | **Group** | **n** | **Age (mean)** | **Male %** | **Female %** | **Diabetes %** | **HTN %** | **CHA₂DS₂-VASc** | **HAS-BLED ≥3** | |
| --- | --- | --- | --- | --- | --- | --- | --- | --- | --- | --- |
| **Ha et al.** | Rivaroxaban | 27,784 | 74 | 54 | 46 | 30.3 | NR | Mean ~3.5 | 31.3% |  |
|  | Warfarin | 27,784 | 74 | 54 | 46 | 30.3 | NR | Mean ~3.5 | 31.3% |  |
| **Weir** | Rivaroxaban | 781 | 79. | 39.5 | 60.5 | NR | NR | NR | NR |  |
|  | Warfarin | 1,536 | 79.9 | 40.7 | 59.3 | NR | NR | NR | NR |  |
| **Coleman** | Rivaroxaban | 1,896 | 72 | NR | NR | NR | NR | NR | NR |  |
|  | Warfarin | 4,848 | 72 | NR | NR | NR | NR | NR | NR |  |
| **Cheng** | Rivaroxaban | 173 | 75 | 55 | 45 | NR | NR | NR | NR |  |
|  | Warfarin | 3,185 | 69 | 51 | 49 | NR | NR | NR | NR |  |

**Table 2: Baseline Characteristics of Included Studies**

| **Author** | **Year** | **Design** | **Country** | **AF Population** | **CKD Stage** | **Sample Size** | **Rivaroxaban (n, Dose)** | **Warfarin (n)** | **Bleeding Definition** | **Stroke/SE Definition** | **Follow-up period** |
| --- | --- | --- | --- | --- | --- | --- | --- | --- | --- | --- | --- |
| Coleman | 2019 | Retrospective | United States | NVAF (OAC naive) | 4 or 5 or HD | 6,744 | 1,896 (~734 received < 20mg) | 4,848 | Cunningham Algorithm (ICD-10 codes) | ICD-10 codes | 1.4 (0.6–2.7) years (median) |
| Ha | 2023 | Retrospective | Australia and Canada | AF / Atrial Flutter | 2–5 (dialysis excluded) | 5,723 | 685 | 5,038 | ICD-10 codes | ICD-10 codes | 7 years |
| Weir | 2020 | Retrospective cohort | United States | NVAF | 4–5 Dialysis | 2,317 | 781 | 1,536 (match-weighted N=781) | ICD-9/10 codes | ICD-9/10 | RIV: 389 days / WAR: 370 days (mean) |
| Cheng | 2021 | Retrospective cohort | Taiwan | NVAF | 5 Dialysis | 3,358 | 173 (50.8% on 10mg, 38.7% on 15mg, 10.4% on 20mg) | 3,185 | ICD-9 | ICD-9 | RIV: 19.1 mo / WAR: 27.4 mo (mean) |

**CKD stages of included patients**

| **Study** | **Treatment** | **N** | **CKD Stage 3a (eGFR 45-59)** | **CKD Stage 3b (eGFR 30-44)** | **CKD Stage 4 (eGFR 15-29)** | **CKD Stage 5,**  **Non-dialysis (<15)** | **CKD Stage 5, dialysis** |
| --- | --- | --- | --- | --- | --- | --- | --- |
| **Weir** | Rivaroxaban | 781 | Not separated | Not separated | 635 (81.3%) | 29 (3.7%) | 117 (15.0%) |
|  | Warfarin | 1536 | Not separated | Not separated | 1249 (81.3%) | 57 (3.7%) | 230 (15.0%) |
| **Coleman** | Rivaroxaban | 1896 | - | - | 228 (12%) | 1,668 (88%) | |
|  | Warfarin | 4848 | - | - | 582 (12%) | 4266 (88%) | |
| **Cheng** | Rivaroxaban | 173 | - | - | - | - | 173 (100%) |
|  | Warfarin | 3185 | - | - | - | - | 3185 (100%) |
| **Ha et al.** | Rivaroxaban | 9310 | 5498 (19.8%) | 3139 (11.3%) | 611 (2.2%) | 62 (0.2%) | - |
|  | Warfarin | 9308 | 5498 (19.8%) | 3138 (11.3%) | 611 (2.2%) | 61 (0.2%) | - |

# **Rivaroxaban Dosing Pattern Table**

| **Study** | **10 mg N (%)** | **15 mg N (%)** | **20 mg N (%)** | **Note** |
| --- | --- | --- | --- | --- |
| Cheng (n=173) | 88 (50.8%) | 67 (38.7%) | 18 (10.4%) | - |
| Ha et al (n=27784) |  | | | 46% reduced dose overall:  eGFR ≥60: 36%  eGFR 45-59: 53%  eGFR 30-44: 77%  eGFR <30: 86% |
| Coleman (n=1896) | <20mg: 732 (38.7%) | | 1163 (61.3%) | Reduced dose not specified |
| Weir (n=781) | 165 (21.1%) | 469 (60.1%) | 115 (14.7%) | Not documented: 32 (4.1%) |

**GRADE Assessment of Included Studies**

| **Study** | **Study Design** | **Risk of Bias** | **Consistency** | **Directness** | **Precision** | **Overall Quality Grade** |
| --- | --- | --- | --- | --- | --- | --- |
| **Ha et al.** | Retrospective cohort (Large database) | Moderate (PS matching, residual confounding possible) | High (large sample size, consistent with others) | Direct (AF + CKD, real-world) | High (large n) | **Moderate** |
| **Weir** | Retrospective cohort (matched) | Moderate (PS matching, moderate sample size) | Moderate (some variation in comorbidity reporting) | Direct | Moderate (smaller n than Jeffry Ha) | **Moderate** |
| **Coleman** | Retrospective cohort (large, claims) | Moderate (claims data limitations, possible miscoding) | Moderate (some heterogeneity in CKD definitions) | Direct | High (large n) | **Moderate** |
| **Cheng** | Retrospective cohort (similar to Weir) | Moderate (PS matched) | Moderate | Direct | Moderate | **Low** |


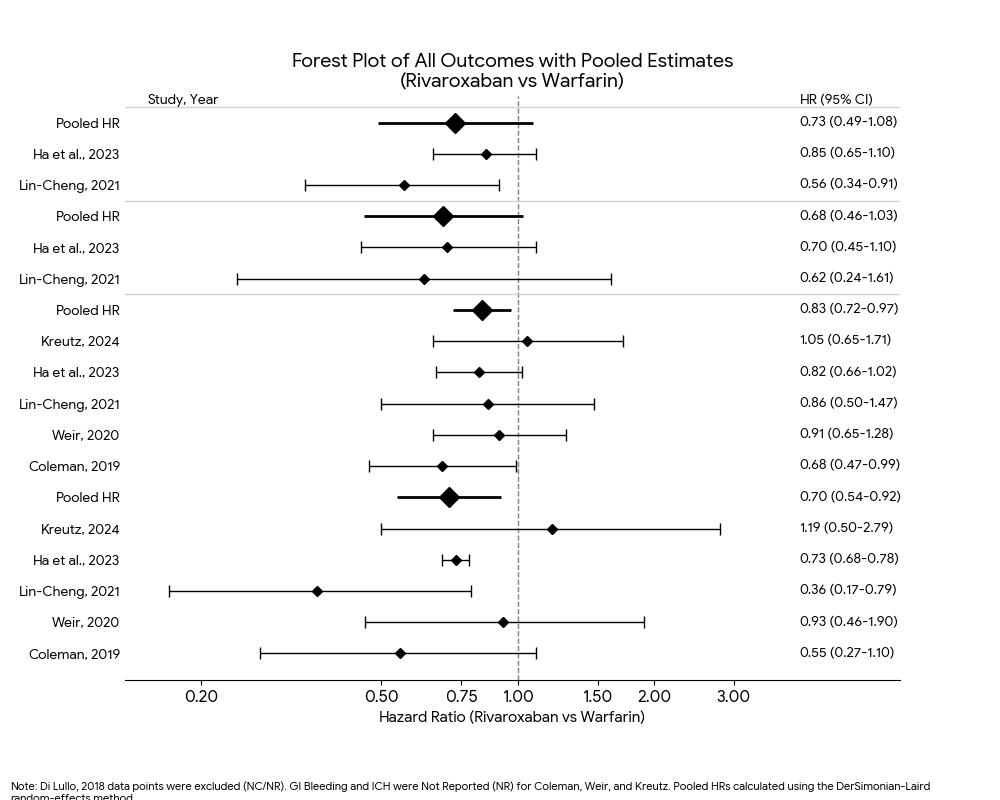

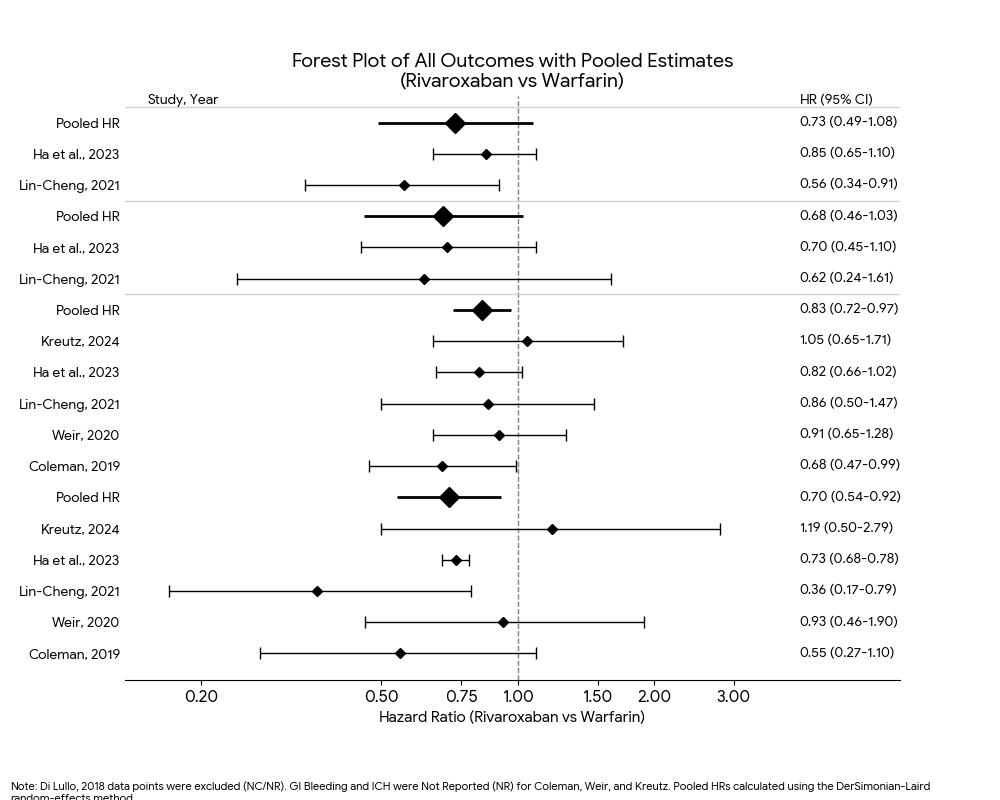

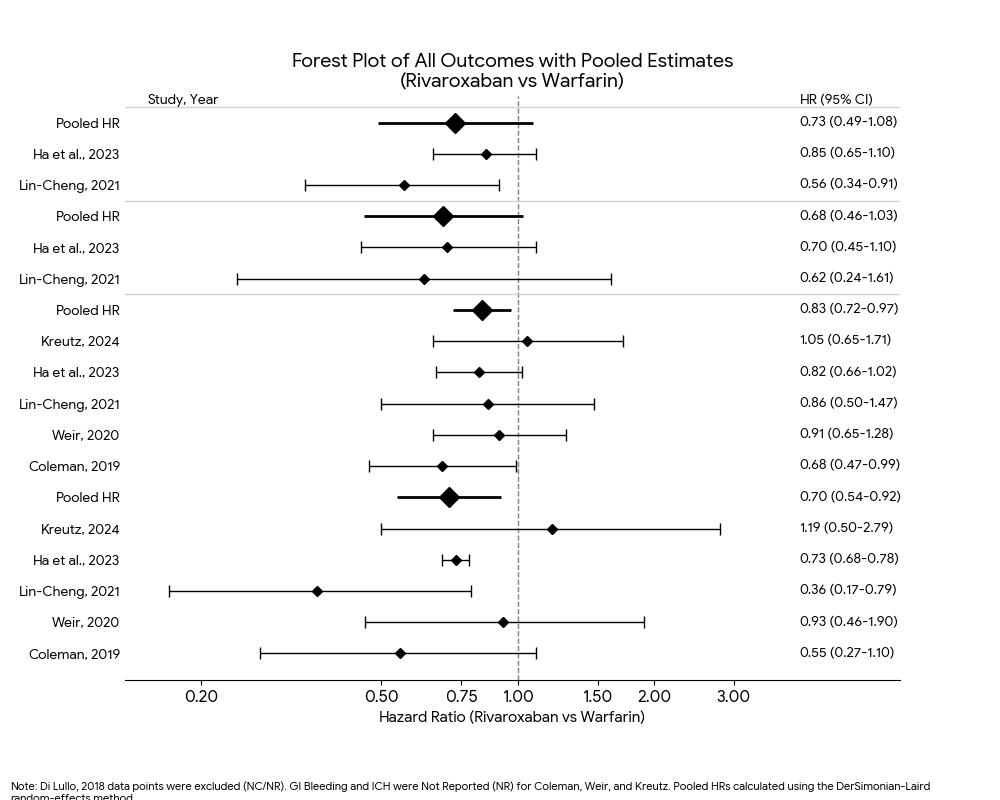

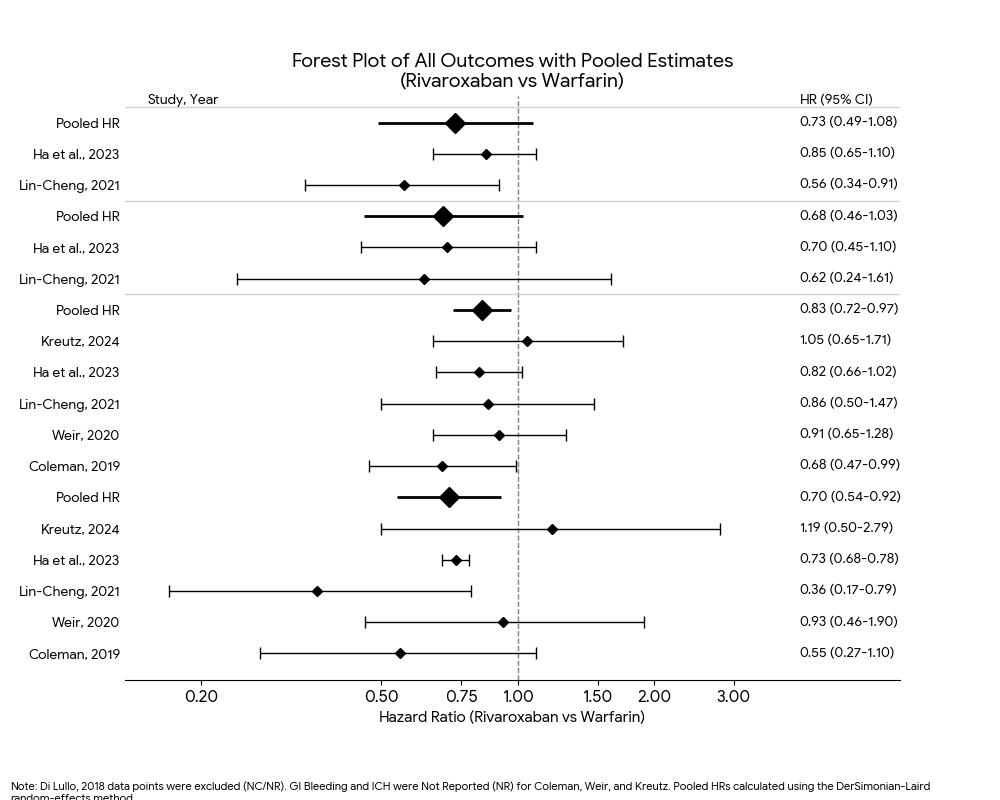


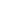

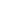

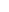

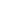

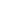

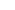

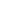

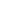

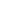

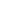

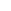

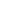

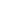

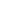

Supplement: Supplementary Data [file mmc1.docx]
